# Serotonin syndrome associated with sertraline, trazodone and tramadol abuse

**DOI:** 10.4103/0019-5545.44913

**Published:** 2009

**Authors:** Neha Nayyar

**Affiliations:** 3323 Circle Brook Dr Apt F Roanoke USA. E-mail: nnayyar@carilion.com

Sir,

To report a case of serotonin syndrome (SS) resulting in a patient with tramadol abuse being on regimen of sertraline, trazodone for major depression and insomnia.

A 55-year-old white man on therapy for major depressive disorder with Zoloft and trazodone since the last 1 year developed sudden delirium, confusion, agitation, tremor, insomnia, diaphoresis, myoclonus, hyperreflexia in lower extremities, mydriasis, tachycardia, and low grade fever with the recent tramadol abuse secondary to worsening knee pain. He had been stable on this medication regimen for 1 year and tramadol was the probable culprit behind the adverse reaction. Battery of lab tests were run on the patient including CBC, BMP, Ammonia level, UDS, BAL, CT Head w/o contrast, MRI Brain, EEG, LP with CSF analysis which were negative. CPK was mildly elevated at 400. He had fever of 100.2 F, fine tremors were present in the upper and lower extremities and myoclonic jerks were noticed. He admitted to tramadol abuse secondary to the worsening knee pain in the last 4-5 days. No evidence of any other co-ingestion. Presumptive diagnosis of Serotonin syndrome was made and the trazodone, sertraline, tramadol were discontinued. Patient required use of chemical restraints on admission secondary to agitation and physical restraints were used for 2 hours due to delirium. Benzodiazepines were given for sedation and control of agitation. He was placed on a cardiac monitor for keeping a watch on the vitals and circulation. His short term memory were impaired and his behavior improved significantly within 24 hours of discontinuation of the offending agents.

SS is a potentially life threatening complication of serotonergic polypharmacy. Considered iatrogenic in presentation, it typically occurs after initiation, dose up-titration or abuse of the offending agent to a regimen including other serotonergic agents. All drugs that directly or indirectly increase central serotonin neurotransmission at postsynaptic 5-HT(1A) and 5-HT(2A) receptors can produce SS. Development of SS also varies from person to person depending on individual vulnerability. It is likely due to the serotonin reuptake inhibition by sertraline and trazodone and (+) enantiomer of tramadol. Tramadol is metabolized to an active metabolite, M1, by the CYP2D6 enzyme.

Clinicians should be aware of the potential for SS when psychotropic and nonpsychotropic agents are coadministered to certain patients.

